# Data on differentially expressed miRNAs in dogs infected with *Leishmania infantum*

**DOI:** 10.1016/j.dib.2018.01.007

**Published:** 2018-01-09

**Authors:** Jaqueline Poleto Bragato, Larissa Martins Melo, Gabriela Lovizutto Venturin, Gabriela Torres Rebech, Leandro Encarnação Garcia, Flavia Lombardi Lopes, Valéria Marçal Felix de Lima

**Affiliations:** a Department of Animal Clinic, Surgery and Reproduction, São Paulo State University (Unesp), School of Veterinary Medicine, Araçatuba, São Paulo, Brazil; b Department of Support, Production and Animal Health, São Paulo State University (Unesp), School of Veterinary Medicine, Araçatuba, São Paulo, Brazil

**Keywords:** Dog, *Leishmania infantum*, miRNA

## Abstract

This paper contains data on differentially expressed miRNAs in peripheral blood mononuclear cells (PBMC) of dogs naturally infected by *Leishmania* (*L.*) *infantum* compared to healthy dogs. In recent years, studies with miRNAs have shown that these molecules play a critical role in the regulation and function of immune response.Differentially expressed miRNAs were identified by microarray, validated by real time PCR and compared with parasite load in the dogs. Targets and pathways were analyzed using the Ingenuity Pathway Analysis program.

**Specifications Table**

**Table:** 

Subject area	*Immunology*
More specific subject area	*Molecular Immunology in canine visceral leishmaniasis*
Type of data	*Table, figure*
How data was acquired	*Blood of dogs infected with L. infantum and healthy dogs were collected by venipuncture and PBMC isolated with Histopaque 1077 gradient (Sigma-Aldrich, St. Louis, MO, USA). DNA was extracted with DNeasy kit (Qiagen, Valencia, California, USA). Total RNA was extracted with mirVana kit with phenol (Life Technologies, USA), microarray was performed using GeneAtlas® Hybridization, Wash, and Stain Kit (Affymetrix, USA). Validation was done by qRT-PCR and analysis in the Ingenuity Pathway Analysis (Qiagen®).*
Data format	*Analysed*
Experimental factors	*Leishmania infantum specie was identified by PCR-RFLP*
Experimental features	*Total RNA was extracted from 5×10*^*6*^*PBMC of dogs infected with L. infantum and healthy dogs. Microarray was performed using miRNAs with RNA Integrity Number greater than 8. Parasite load was measured by qRT-PCR and correlated with miRNA expression.*
Data source location	*São Paulo State University, Araçatuba, São Paulo, Brazil.*
Data accessibility	*Microarray data were deposited in Gene Expression Omnibus with the access number*GSE105443

**Value of the data**

• MicroRNA profile in dogs with VL was described for the first time.
• *Leishmania infantum* infection differentially regulates miRNA expression.
• Due to reemergence of VL, miRNA data obtained herein is fundamental to disease control.

## 1 Data

Data shown in this article provide information about miRNAs differentially expressed in PBMC of dogs with VL compared to healthy dogs. Total RNA was extracted from PBMC of healthy dogs (control group) and infected dogs (infected group). Control group was composed by five healthy dogs of different breeds, weight and age between 1 and 5 years. Infected group was composed by ten dogs naturally infected with *L. infantum* with at least 3 characteristic symptoms of CVL, housed in Araçatuba, an endemic region in Brazil. All animals were of different breeds, weight and age between 1 and 5 years. Physical examination, complete blood count and serum biochemical profile were performed. ELISA was employed to confirm the presence or absence of anti-*Leishmania* antibodies, considering a cut-off in 0.285 [1] (Table 1).

**Table 1:** Optical density on ELISA and clinical signs of animals naturally infected and control healthy.

Animal	ELISA	Clinical Signs
Infected 1	1.333	Onychogryphosis, skin lesions, alopecia, ear lesions, anemia, hepatosplenomegaly
Infected 2	0.511	Onychogryphosis, cachexia, skin lesions, alopecia, ear lesions, anemia, hepatosplenomegaly
Infected 3	0.974	Onychogryphosis, cachexia, ear lesions, anemia
Infected 4	1.283	Onychogryphosis, alopecia, ear lesions, anemia
Infected 5	0.356	Lymphadenopathy, onychogryphosis, skin lesions, hepatosplenomegaly
Infected 6	1.323	Onychogryphosis, skin lesions, ear lesions, anemia
Infected 7	1.315	Onychogryphosis, cachexia, skin lesions, ear lesions, anemia
Infected 8	0.907	Onychogryphosis, cachexia, ear lesions, anemia, hepatosplenomegaly
Infected 9	1.089	Lymphadenopathy, onychogryphosis, skin lesions, alopecia, ear lesions, anemia, hepatosplenomegaly
Infected 10	0.711	Onychogryphosis, alopecia, ear lesions, anemia
Control 1	0.026	No clinical sings
Control 2	0.032	No clinical sings
Control 3	0.028	No clinical sings
Control 4	0.049	No clinical sings
Control 5	0.055	No clinical sings

## 2 Experimental design, materials and methods

### 2.1 Animal screening and collection of samples

This study was approved by the Brazilian Council on Animal Experimentation (COBEA), with the approval of the Committee for Ethics in Animal Use (CEUA) of UNESP – Universidade Estadual Paulista "Júlio de Mesquita Filho" - Campus de Araçatuba - School of Veterinary Medicine - FMVA (process 00978/2016).

Five healthy animals upon clinical examination, complete blood count and serum biochemical profile were selected for this study, and ten animals naturally infected with *Leishmania infantum,* from the Zoonosis Control Center of Araçatuba, were selected. These animals contained at least three characteristic clinical signs of the disease, including onychogriphosis, weight loss, ear-tip injuries, periocular lesions, alopecia, skin lesions and lymphadenopathy. Infected animals were classified in the clinical stage II of the disease [2]. Blood from both groups (infected and control) was collected in tubes without EDTA to obtain serum for biochemical profile and perform indirect ELISA assay for the detection of anti-leishmania antibodies [3], and in EDTA tubes for hemogram and isolation of the PBMC. Infected animals were euthanized by barbiturate anesthesia (Tiopental, Cristália Itapira, SP), followed by intravenous injection of potassium chloride 19.1% by the same route, in compliance with local laws.

### 2.2 Isolation of peripheral blood mononuclear cells

PBMC were isolated by gradient Histopaque® 1077 (Sigma-Aldrich, St. Louis, MO, USA) following manufacturer's instructions. Cells were then washed twice in phosphate buffered saline solution at pH 7.2. After isolation, these cells were counted in a Neubauer chamber prior to extraction of DNA and total RNA.

### 2.3 DNA extraction and determination of the Leishmania species

DNA extraction from PBMC samples from the experimental dogs was performed using 5×10^6^ cells with the commercial DNAeasy® kit (Qiagen, Valencia, California, USA) according to manufacturer's recommendations. Extracted DNA was quantified in spectrophotometer 260/280 (NanoDrop, Thermo Fisher Scientific) for evaluation of purity and then stored at −20 °C until analysis.

Determination of *Leishmania* species was performed by PCR-RFLP (Restriction Fragment Length Polymorphism) [4], comparing the restriction profile of the sample with obtained from *L. infantum* (IOC / L0575-MHOM / BR / 2002 / LPC-RPV), *L. braziliensis* (IOC / L0566-MHOM / BR / 1975 / M2903) and *L. amazonensis* (IOC / L0575-MHOM / BR / 1967 / PH8) as positive controls and water as negative control.

### 2.4 Quantification of parasite load by real-time PCR

**Fig. 1:**
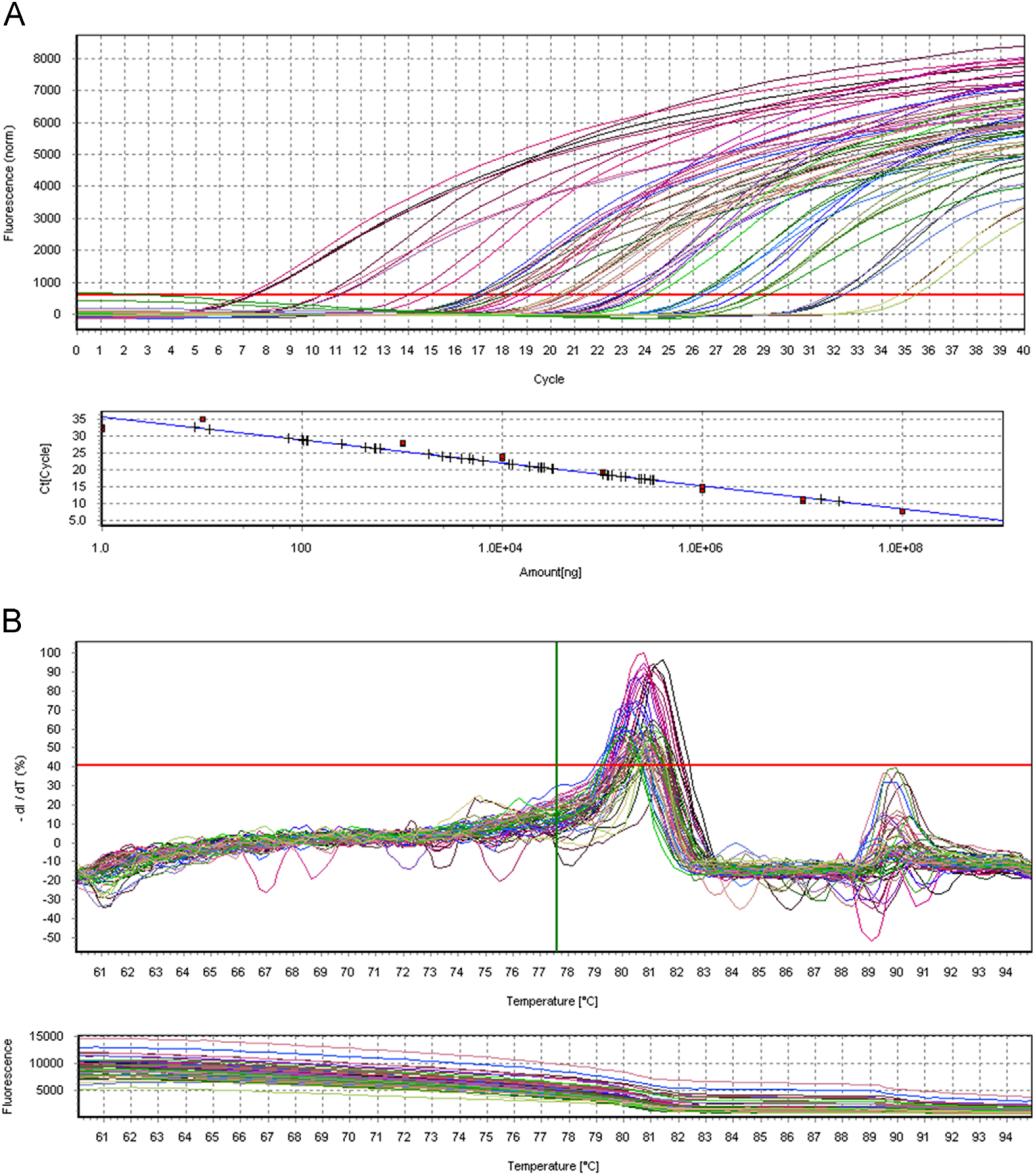
Real Time PCR for quantification of parasite load. Amplification and quantification of DNA (A) and melting curve (B).

Parasite load quantification was performed by real-time PCR using the SYBR Green PCR Master Mix (Applied Biosystems®) in a final reaction volume of 20 μL. Specific primers (5 'CCTATTTTACACCAACCCCCAGT 3' and 5 'GGGTAGGGGCGTTCTGCGAAA 3'), amplifying a 116 bp fragment of the kinetoplast DNA (kDNA) of *Leishmania* spp., at a concentration of 900 nM [5], and 50ng of sample DNA were used. Amplification condition used was an initial heating of 95 °C for 10 min, followed by 40 cycles of 95 °C for 15 s and 65 °C for 60 s. Upon the end of amplification, a dissociation curve of the amplified fragment was determined from 60 °C to 95 °C with an increase of 0.5 °C every 5 s. A standard curve with DNA from *Leishmania infantum* promastigotes (MHOM/BR00/MER02) with dilution of 10^8^ to 10^1^ ng of parasite DNA was performed for each reaction (Fig. 1).

### 2.5 Extraction and quantification of total RNA

Extraction of total RNA from PBMC was performed on the samples with the commercial mirVana® kit for isolation with phenol (Life Technologies, USA), which preserves small RNAs, following procedure indicated by the manufacturer. After isolation, total RNA was stored at −80 °C until evaluation of quality and concentration.

**Fig. 2:**
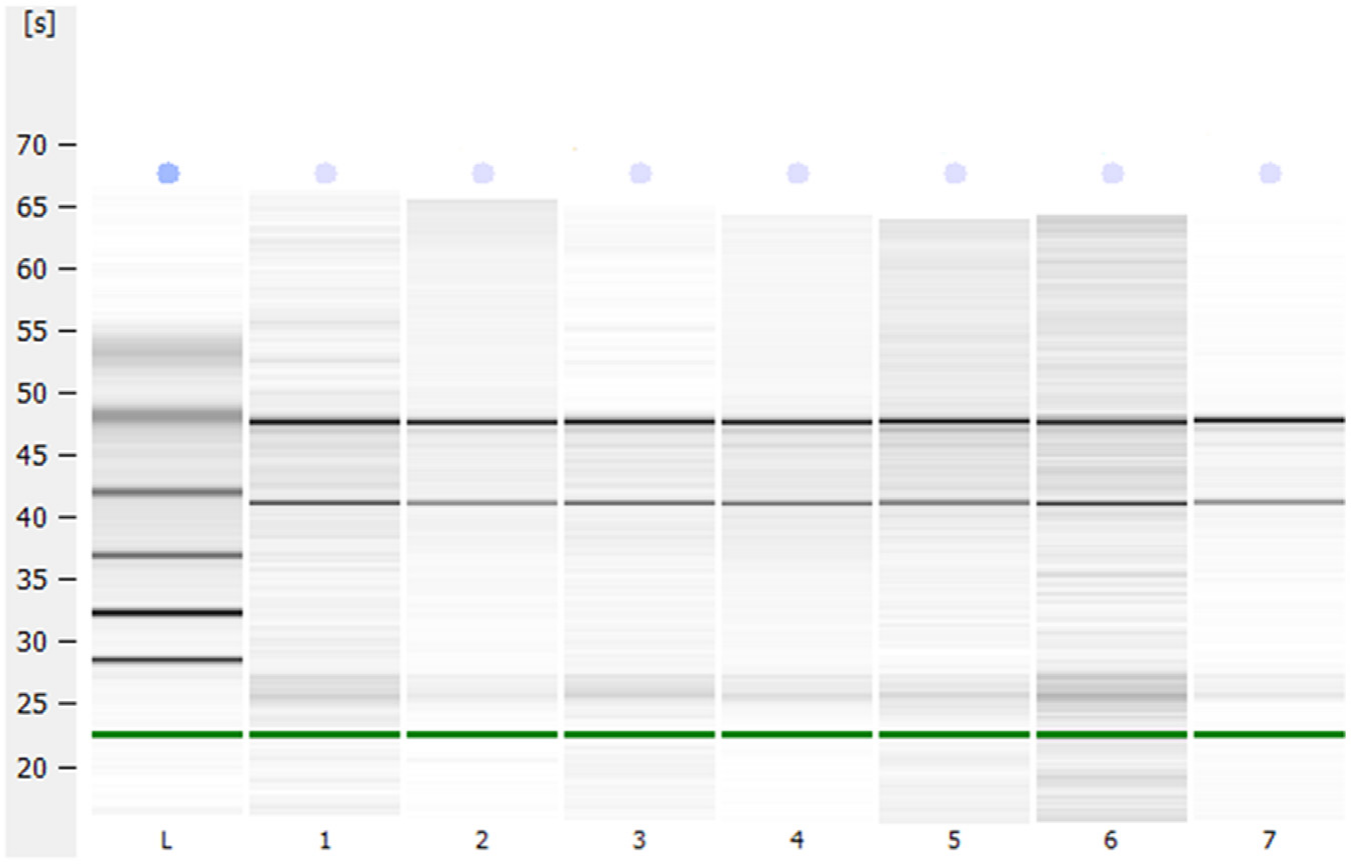
Quality of miRNA. Bioanalyzer gel image of RNA samples used for microarray analysis. L (Ladder) Each number represents the sample of one animal. RNA Integrity Number (RIN) is respectively 9.1 (1), 10(2), 10 (3), 9.7 (4), 10(5), 8.8(6) and 9.4(7) in the seven samples illustrated.

Isolated RNAs were analyzed by spectrophotometry (NanoDrop, Thermo Fisher Scientific) for evaluation of their quantity (260/280). Before performing microarray, samples were also analyzed for RNA quality by capillary electrophoresis (Bioanalyzer, Agilent Technologies, USA) using the commercial Agilent RNA 6000 Nano kit, following manufacturer's instructions (Fig. 2).

### 2.6 Microarray

Total RNA samples with concentration above 30 ng/µl and quality (RIN>8) were used to perform microarray analysis using a miRNA 4.1 Array Strip (Affymetrix, USA), containing probes designed for the miRNAs of several species, including 291 miRNA probes of *Canis familiaris*.

MicroRNAs were biotinylated using the Affymetrix FlashTag™ Biotin HSR RNA Labeling Kit following manufacturer's instructions. For the microarray, GeneAtlas® Hybridization, Wash, and Stain Kit for miRNA array Strips were used, following manufacturer's instructions.

Microarray data were deposited in the Gene Expression Omnibus with the accession number GSE105443 according to the minimum information about microarray experiment (MIAME) standards.

### 2.7 Microarray data analysis

**Fig. 3:**
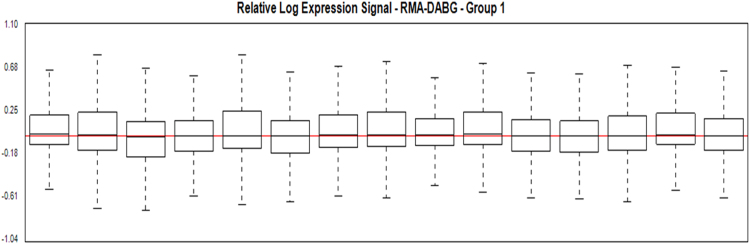
Values of relative log expression signal (RMA-DABG normalized) between groups.

**Fig. 4:**
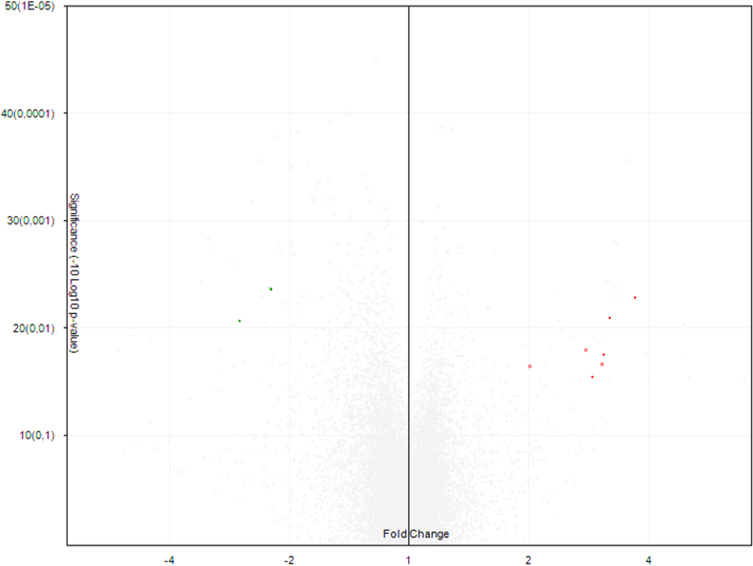
Distribution of differentially expressed miRNAs. Upregulated (red) and downregulated (green) miRNAs in PBMC of dogs infected with *L. infantum* compared to healthy dogs.

Normalization and quality of microarray analysis of the miRNAs of control and infected dogs were performed in the Expression Console Software program, version 1.4.1 (Affymetrix, Thermo Fisher Scientific, USA) (Fig. 3). Differential analysis of the miRNAs was performed in the Transcriptome Analysis Console (Affymetrix, Thermo Fisher Scientific, USA) (Fig. 4).

Analysis of targets and pathways of the miRNAs differentially expressed in dogs with VL were performed using the Ingenuity Pathway Analysis (Qiagen, Redwood City, CA, USA).

### 2.8 Real-time PCR for miRNA analysis

**Fig. 5:**
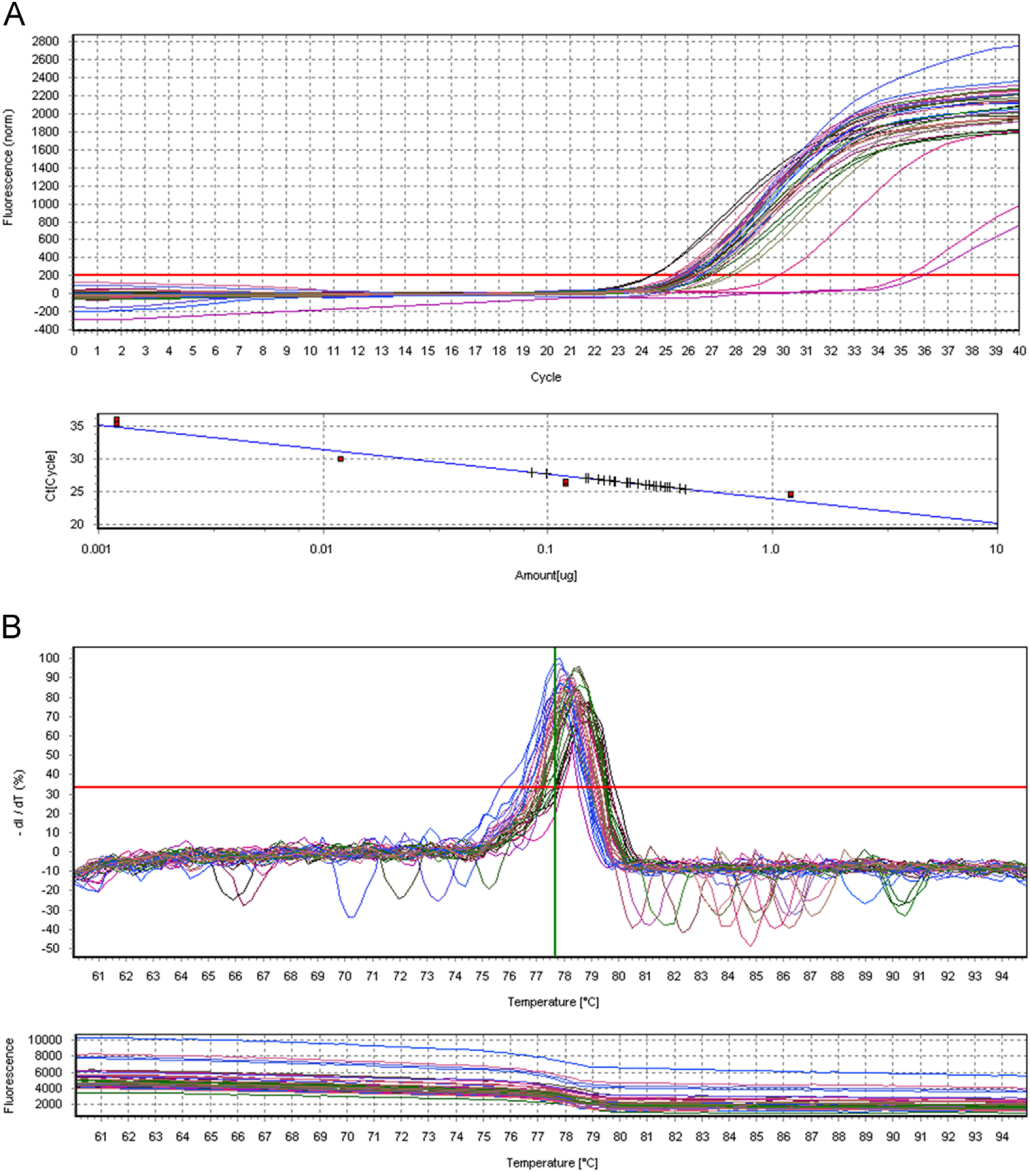
Validation of miRNAs by Real time PCR. Amplification and quantification of miRNA (A) and melting curve (B).

To validate the results obtained by microarray, real-time PCR (qPCR) was performed. cDNA production was performed using the miScript RT II kit (Qiagen™), as recommended by the manufacturer. qPCR reactions were performed using commercially available specific primers for dog miRNAs of interest and reference genes (miScript, Qiagen™) using the SYBR Green system (myScript SYBR Green PCR kit, Qiagen™) in the real-time thermal cycler (RealPlex, Eppendorf™). Amplification conditions were determined by the manufacturer. For each reaction, a standard curve with serial dilution of a pool of cDNAs was performed. MicroRNA expression was calculated based on the values of the standard curve, by the quotient of the target miRNA value and the housekeeping gene value. Fig. 5a and b presents one of the validated miRNAs in qPCR.

### 2.9 Statistical analysis

Statistical analyses were performed using the GraphPad Prism 6 software (GraphPad Software, Inc., La Jolla, CA, USA). Analysis of variance (ANOVA) was performed for treatment comparison in the microarray. Mann-Whitney test was performed to validate the real-time PCR values of the miRNAs. Spearman correlation was made to evaluate association between parasite load and miRNA expression. Fisher test was used in the IPA program for canonical pathway analysis. Results were considered significant when *p*<0.05.
